# Unravelling mechanisms of bacterial recognition by Acanthamoeba: insights into microbial ecology and immune responses

**DOI:** 10.3389/fmicb.2024.1405133

**Published:** 2024-08-23

**Authors:** Fauzy Nasher, Brendan W. Wren

**Affiliations:** Department of Infection Biology, London School of Hygiene and Tropical Medicine, London, United Kingdom

**Keywords:** Acanthamoeba, pattern recognition receptors, microbe-associated molecular patterns, phagocytosis, immune recognition, flagellin, lipopolysaccharides, lipoteichoic acid

## Abstract

Acanthamoeba, are ubiquitous eukaryotic microorganisms, that play a pivotal role in recognizing and engulfing various microbes during predation, offering insights into microbial dynamics and immune responses. An intriguing observation lies in the apparent preference of Acanthamoeba for Gram-negative over Gram-positive bacteria, suggesting potential differences in the recognition and response mechanisms to bacterial prey. Here, we comprehensively review pattern recognition receptors (PRRs) and microbe associated molecular patterns (MAMPs) that influence Acanthamoeba interactions with bacteria. We analyze the molecular mechanisms underlying these interactions, and the key finding of this review is that Acanthamoeba exhibits an affinity for bacterial cell surface appendages that are decorated with carbohydrates. Notably, this parallels warm-blooded immune cells, underscoring a conserved evolutionary strategy in microbial recognition. This review aims to serve as a foundation for exploring PRRs and MAMPs. These insights enhance our understanding of ecological and evolutionary dynamics in microbial interactions and shed light on fundamental principles governing immune responses. Leveraging Acanthamoeba as a model organism, provides a bridge between ecological interactions and immunology, offering valuable perspectives for future research.

## Introduction

Microbial interactions are one of the main driving forces governing ecosystem dynamics, influencing nutrient re-cycling and even disease transmission. Among the diverse cast of microorganisms, Amoebozoa, the phylum closest to fungi and animals demonstrate remarkable versatility in their relationship with other microorganisms. Key representatives from the genera are Acanthamoeba (see glossary) and Dictyostelids (Thewes et al., 2019).

Acanthamoeba, are free-living ubiquitous unicellular eukaryotes, and stand out as masters of microbial interactions (Rayamajhee et al., 2022). Beyond their ecological role, Acanthamoeba are significant in medical contexts as they can cause serious infections such as keratitis, an eye infection that can lead to vision loss (Rayamajhee et al., 2021b; Zhang et al., 2023), and granulomatous amoebic encephalitis, a fatal infection of the central nervous system (Das et al., 2020). Understanding Acanthamoeba is thus crucial for both environmental and human health considerations.

The study of Acanthamoeba-bacteria interactions has evolved significantly over the years. Historically, research began with the characterization of Acanthamoeba as free-living amoebae with a unique predatory role in the environment, these early studies focused on their general biology and ecological impact. As research progressed, the interest shifted toward understanding their interactions with bacteria, particularly due to the amoebae’s complex predatory behavior and their role in microbial ecology. This body of research has expanded to explore the genetic and molecular mechanisms underlying these interactions, highlighting the evolutionary and ecological significance of these processes (Anderson, 2018; Shi et al., 2021, 2024).

Acanthamoeba serve as valuable model organisms for studying host-pathogen interactions due to their predatory phagocytic nature, resembling that of more complex immune cells like macrophages (Khan and Siddiqui, 2014; Price Christopher et al., 2024). Their interactions with other microorganisms have been shown to result in lateral gene transfer (LGT) between Acanthamoeba and their prey (Clarke et al., 2013). The first whole genome assembly of *Acanthamoeba castellanii* Neff strain (ATCC 30010) revealed ~450 genes (20%) of the 15,455 compact intron-rich genes had been co-opted into its genome through inter-kingdom LGT. This likely enabled Acanthamoeba adaptation to diverse environments which is evident by the diversity of pattern recognition receptors (PRRs); elemental receptors that recognize specific molecular structures on the surface of microbes (Li and Wu, 2021). Many Acanthamoeba PRRs share predicted orthologous functions with innate immune phagocytes in higher eukaryotes like monocytes, neutrophils, and macrophages (Li and Wu, 2021). Acanthamoeba and prey interactions is a complex process that hinges on microbe-associated molecular patterns (MAMPs) on the surfaces of microorganisms (Newman et al., 2013). The innate immune response, originating from ancient evolutionary processes (Kubelkova et al., 2023), features defined receptors for MAMPs, mitogen-associated protein kinase (MAPK) activation, and antimicrobial peptide production (Aweya et al., 2022). These shared characteristics may have evolved divergently from an ancestral unicellular organism predating the divergence of life kingdoms (Weiss et al., 2016).

Acanthamoeba preferentially feed on Gram-negative bacteria (Bottone et al., 1994; Khan and Siddiqui, 2014; Rayamajhee et al., 2021a, 2023). This preference may derive from the multitude of receptors encoded in the genome of *A. castellanii* Neff strain that may facilitate recognition and subsequent predation. This recognition potential likely arise from the unique surface structure and biochemical composition of Gram-negative bacterial cell walls compared to Gram-positive bacteria. Interestingly, interactions between Acanthamoeba and *Listeria monocytogenes*, a Gram-positive bacterium distinguished by its capability to invade eukaryotic cells (Bierne and Cossart, 2002; Cossart and Toledo-Arana, 2008), have been reported to be random encounters, where phagocytosis occurs as a consequence of trapped cells (de Schaetzen et al., 2022). In contrast, interactions with the Gram-negative bacteria *Campylobacter jejuni* depend on glycans decorating the flagella, a cell surface appendage (Nasher et al., 2018). The implications of this interactions for microbial ecology and immune responses are significant and these interactions can influence the transmission and pathogenicity of bacterial infections, potentially increasing the risk of disease outbreaks (Rayamajhee et al., 2024a,b).

Given these observations, a comprehensive review of Acanthamoeba’s interactions with various bacteria is warranted, particularly focusing on the recognition mechanisms of cell surface structures. This review highlights similarities and differences between Acanthamoeba and immune cells of warm-blooded hosts. Understanding these interactions, influenced by bacterial cellular components, holds broad implications for microbial ecology, evolutionary dynamics, and host immune responses, contributing to the ecology and evolution (eco-evo) perspective of pathogenomics (Pallen and Wren, 2007). This review is aimed to set the scene for future research by providing an overview of the intricate interactions between Acanthamoeba and bacteria.

## A multifaceted approach to recognition and interaction

Recognition of bacterial surface structures adorned with glycans is crucial for phagocytic cells like macrophages and neutrophils, as it forms a central aspect of the immune response against invading microorganisms [detailed review available here (Prado Acosta and Lepenies, 2019)]. Post-translational modifications of bacterial structures, specifically through glycosylation, act as key MAMPs for phagocytes, underscoring the significance of bacterial glycans in host recognition. These carbohydrate patterns exhibit considerable variability among different cell types, prompting phagocytes to evolve mechanisms to distinguish between self and non-self. Similar to phagocytic cells in higher organisms, Acanthamoeba also recognize and respond to bacterial glycans (Nasher and Wren, 2023), highlighting the conserved nature of glycan-mediated mechanisms across different organisms and the adaptive benefits of recognizing these molecules (van Kooyk and Rabinovich, 2008; Prado Acosta and Lepenies, 2019).

## Post-translational modification of bacterial cell surface structures as MAMPS

These carbohydrate patterns on MAMPS include common eukaryotic sugars such as glucose, galactose, *N*-acetylglucosamine, *N*-acetylgalactosamine, and sialic acid, along with a diverse array of unique bacterial sugars. Bacterial glycans feature rare deoxy amino sugars, including pseudaminic acid, legionaminic acid, d-bacillosamine, d-2,4-diacetamido-2,4,6-trideoxygalactose (DATDG), d- and l-*N*-acetylfucosamine (l-FucNAc), l-*N*-acetylrhamnosamine (l-RhaNAc), l-*N*-acetylpneumosamine (l-PneNAc), and l-*N*-acetylquinovosamine (l-QuiNAc) (Barrett and Dube, 2023). These sugars may vary based on microbial type and environmental conditions, further influencing host pathogen interactions.

This review focuses on bacterial cell surface appendages and their PTM with glycans, which are critical for their recognition and subsequent phagocytosis by Acanthamoeba. Flagellin is the protein subunit of bacterial flagella, essential for motility and recognized by immune receptors such as Toll-like receptor 5 (TLR5) (Nedeljković et al., 2021). Lipopolysaccharide (LPS), a major component of the outer membrane of Gram-negative bacteria, consists of lipid A, core polysaccharide, and O antigen, and is a potent endotoxin recognized by TLR4 (Mazgaeen and Gurung, 2020). Lipoteichoic Acid (LTA), found in the cell walls of Gram-positive bacteria, plays a role in cell wall maintenance and is recognized by TLR2 (Li and Wu, 2021). Capsular Polysaccharides (CPS), which are polysaccharide layers surrounding some bacteria, provide protection against phagocytosis and environmental stresses and are recognized by specific receptors on phagocytic cells (Gao et al., 2024). These insights underscore the ancient origins and fundamental importance of glycan recognition in immune defense mechanisms.

## Mannose-binding proteins (MBPs)

Acanthamoeba employ a sophisticated array of molecular players to interact with prey, including C-type lectins. C-type lectins have major functional roles of innate and adaptive immune responses (Kalia et al., 2021). These proteins are characterized by carbohydrate-recognition domains (CRDs), which exhibit calcium-dependent binding with specific carbohydrate ligands (Weis and Drickamer, 1994). The genome of *A. castellanii* Neff strain encodes two distinct mannose-binding proteins (MBPs), MBP1 (Q6J288) and MBP2 (L8GXW7), which share a conserved DUF 4114 domain (DUF-domain of unknown function) (Corsaro, 2022). Positioned on the surface membrane, Acanthamoeba MBPs are mannose-containing glycoproteins, lacking N-acetyl-galactosamine (GalNac) but modified by *N*-linked glycosylation (Garate et al., 2005).

MBPs bind to carbohydrates with hydroxyl groups at specific positions on the pyranose ring (Feinberg et al., 2000; Ng et al., 2002). Consequently, MBPs exhibit pronounced affinity for ligands like mannose and GlcNAc. While MBPs generally show minimal binding affinity to carbohydrates with incompatible steric arrangements like galactose, certain contexts, such as specific structural conformations or modifications, may still facilitate interactions with carbohydrates like sialic acid (Weis and Drickamer, 1994). For example, interactions with bacterial components that are composed of sialic acid polymers might involve other receptors or co-factors that enhance binding affinity. This steric precision coupled with variations in the spatial arrangement of its ligands, MBPs facilitate the selective identification of carbohydrates structures on the surface of microorganisms (Veldhuizen et al., 2011; Paurević et al., 2024). While the exact molecular targets of MBPs on bacterial surfaces remain elusive, its affinity for carbohydrates possessing hydroxyl groups at the 3- and 4-positions of the pyranose ring implies potential interactions with a range of microbial components. These include lipopolysaccharides LPS (Raetz and Whitfield, 2002) on Gram-negative bacteria, CPS (Azurmendi et al., 2020), and LTA on Gram-positive bacteria (Zhang et al., 2006). Functionally versatile, Acanthamoeba MBPs serve as receptors for a spectrum of pathogens, including *Legionella pneumophila* (Declerck et al., 2007) and *Escherichia coli* (Carvalho et al., 2003). The recent availability of more robust Acanthamoeba genome sequences (Sharma et al., 2022) have enabled a deeper understanding of Acanthamoeba MBPs mechanisms and interactions, shedding further light on its divergence that lead to variations in bacterial interactions efficiency (Matthey-Doret et al., 2022).

## d-Galactoside/l-Rhamnose-binding lectins (RBLs)

*A. castellanii* Neff strain genome also revealed the presence of 11 d-galactoside/l-Rhamnose-binding lectins (RBLs) domain-containing proteins which are absent in other Amoebozoa (Clarke et al., 2013). Similarly to MBPs, RBLs play pivotal roles as PRRs, aggregating bacteria by interacting with LPS and LTA (Subi and Shabanamol, 2022; Wang et al., 2023). Notably, carbohydrate binding RBLs are structurally similar to sea urchin egg lectins (SUEL) (Sugawara et al., 2020) and have a higher affinity for smooth LPS than they do for rough LPS variants (Tateno et al., 1998; Tateno, 2010), highlighting the significance of the O-antigen in host responses.

## Role of O-antigen in bacterial recognition

The O-antigen is a crucial component found in both LPS and bacterial capsules. LPS, a well-studied virulence factor in Gram-negative bacteria. LPS is composed of three distinct domains dictating its biological and immunological properties (Bertani and Ruiz, 2018). LPS serves as a key determinant in bacterial recognition by Acanthamoeba (Tezcan-Merdol et al., 2004). Interestingly, while certain bacterial serotypes may influence the efficiency of bacterial consumption (Liu and Koudelka, 2023a), recent studies suggest that Acanthamoeba recognizes the more conserved components of LPS, such as the core OS region, for detection and phagocytosis (Arnold et al., 2016; Liu and Koudelka, 2023b). This ability is reminiscent of dendritic cells recognition of LPS core OS by DC-SIGN (Dendritic Cell-Specific Intercellular adhesion molecule-3-Grabbing Non-integrin), a mannose-binding protein that mediates phagocytosis (Svajger et al., 2010). Additionally, monoclonal antibodies against the core OS of various bacteria can bind this feature in the intact membrane, suggesting that its proximity does not block the ability to be bound by proteins (Shenep et al., 1987; Bos et al., 2004). This interaction is characteristic of the eco-evo dynamic and sheds light on the co-evolution of bacteria and protists in their natural environment.

A recent study using strains of *E. coli* that share the same inner core sugars demonstrated that the Kdo2 moiety within the inner core of LPS is necessary and adequate for *E. coli* recognition and internalization by *A. castellanii* (Liu and Koudelka, 2023b). Also, the specific composition of the variable outer core region influences the efficiency of consumption by *A. castellanii*. This suggests that Acanthamoeba may sense and recognize the more conserved components of LPS, and it is quite likely that rough-form LPS is also recognized by Acanthamoeba similarly. Intriguingly, it was proposed that Lipid A possibly evolved in response to persistent interactions with protists in the natural environment, rather than the occasional infective interactions in humans and animals (Liu and Koudelka, 2023b).

In studies on *Salmonella enterica* serovars, variations in their internalization by Acanthamoeba have been observed. Serovars such as *S. enterica* Dublin are internalized more efficiently compared to others like *S. enterica* Enteritidis or Typhimurium (Tezcan-Merdol et al., 2004). This differential recognition and internalization efficiency can be attributed to variations in the O-antigen, which often contains mannose (Reeves et al., 2013), underscoring the importance of this component in bacterial-protozoan interactions. The preference of Acanthamoeba for specific *Salmonella* serogroups suggests an adaptive mechanism in which these protists have evolved to recognize and differentiate between bacterial strains based on their O-antigen (Wildschutte et al., 2004). This dynamic interaction further illustrates the ongoing evolutionary arms race between bacteria and their protistan predators.

## Capsular polysaccharides (CPS) and their role in immune evasion

The O-antigen is also a key component of bacterial CPS. CPS protects bacteria from phagocytes due to their hydrophilic and negatively charged properties (Cress et al., 2014), this creates repulsive forces when they encounter phagocytes, hindering interaction (Whitfield et al., 2020). *E. coli* K1 fulfills this paradigm (Arredondo-Alonso et al., 2023), however, the K1 CPS presents a unique scenario when interacting with Acanthamoeba.

Mutations to *neuDB* gene cluster, which is essential for CPS production, lead to reduced internalization and decreased survival of *E. coli* K1 within Acanthamoeba (Jung et al., 2007). This indicates that the capsule is crucial for Acanthamoeba interactions and intracellular survival. Interestingly, Acanthamoeba interactions with the K1 capsule, which contains a polymer of alpha−2,8-linked N-acetylneuraminic acid (sialic acid) (Arredondo-Alonso et al., 2023), is facilitated by specific receptors or mechanisms that can recognize sialic acid. This surprising interaction highlights Acanthamoeba ability to respond to different carbohydrates on CPS, which leads to phagocytosis.

CPS likely also provides protection to the bacteria from the amoebae. This phenomenon was recently illustrated in *Klebsiella pneumoniae*, where interactions with *A. castellanii* led to *K. pneumoniae* CPS hypermucoviscosity (Huang et al., 2023). CPS molecular compositions vary extensively even within strains of the same species, with some species from different orders producing identical structures (Whitfield et al., 2020). The presence of highly similar machinery responsible for producing identical polysaccharides in different microbes suggests that capsular gene clusters may have been acquired through LGT or functional convergent evolution (Whitfield et al., 2020). Understanding these dynamics is crucial for elucidating the intricate mechanisms underlying Acanthamoeba interactions with bacteria, as CPS structures likely play a significant role in prey recognition and predation preferences.

## Interaction with lipoteichoic acid (LTA)

In contrast to LPS and CPS, there has only been a single report investigating Acanthamoeba interactions with LTA (52). Like LPS, *A. castellanii* was shown to readily respond to LTA from *Staphylococcus aureus* as an attractant. This interaction may involve recognition of glycosylated components of the glycerolphosphate polymer, as some studies suggest glycosylation of the hydroxyl component of glycerolphosphate allows for lectin-like receptor binding (Weidenmaier and Peschel, 2008; Percy and Gründling, 2014). However, at high concentration LTA was reported to be toxic to the amoebae and act as a repellent (Schuster and Levandowsky, 1996). While both LTA and LPS share pathophysiological properties (Percy and Gründling, 2014), it is noteworthy that they possess distinct chemical structures and originate from different bacterial types. LTA, found predominantly in Gram-positive bacteria, differs structurally from LPS, the hallmark component of Gram-negative bacterial outer membranes. These structural disparities may underlie variations in their interactions with Acanthamoeba and further investigation into how these structural differences influence Acanthamoeba behavior could provide insights into their microbial preferences and ecological roles.

## Secreted immune-related proteins

Acanthamoeba also possess a diverse array of predicted secreted immune-related proteins, including bactericidal/permeability-increasing proteins (BPIs) and lipopolysaccharide-binding proteins (LBPs), which may play crucial roles in host defense mechanisms (Clarke et al., 2013). Six members of the BPI/LBP-like family proteins (ACA1_147410, ACA1_212480, ACA1_238450, ACA1_374090, ACA1_388520, and ACA1_388570) have been identified in Acanthamoeba, characterized by the presence of N-terminal signal peptides, suggesting their secretory nature and potential binding to hydrophobic ligands. In vertebrates, BPI and LBP are key components of the innate immune system, recognizing and responding to bacterial infections, particularly those caused by Gram-negative bacteria LPS (Theprungsirikul et al., 2021). While mammalian LBP exhibits direct binding to the outer membrane of Gram-negative bacteria and facilitates endotoxin conveyance, BPI demonstrates a strong affinity for unmodified endotoxin aggregates, isolated outer membranes (OM), and intact, live Gram-negative bacteria, thereby preventing harmful immune hyperactivation in the host (Krasity et al., 2011; Bülow et al., 2018). However, it remains to be investigated whether similar mechanisms exist in Acanthamoeba. In addition, *A. castellanii* genome also encodes a membrane-bound Myeloid Differentiation factor 2 (MD-2) protein. In mammalian cells, MD-2 is required for LPS signaling of TLR4 and opsonophagocytosis (Shimazu et al., 1999). LPS is extracted from the bacterial membrane by LBPs and transferred to MD-2, LPS binds to the hydrophobic cavity of this protein, and this interaction directly mediates TLR4/MD-2 complex homodimerization and results in downstream signaling in vertebrates (Shimazu et al., 1999). Acanthamoeba lack TLR4, therefore the role of MD-2 in sensing and phagocytosis remains unclear. We therefore speculate that MD-2 activation in Acanthamoeba could enhance phagocytosis of bacterial cells.

## Peptidoglycan recognition proteins (PGRPs)

The *A. castellanii* Neff strain genome sequence also identified two peptidoglycan recognition proteins (PGRPs), which share structural similarities with bacteriophage T7 lysozyme and are conserved across various species, from insects to mammals (Clarke et al., 2013; Bastos et al., 2020). PGRPs serve as PRRs, binding or hydrolyzing peptidoglycans found on both Gram-negative and Gram-positive bacteria (Bastos et al., 2020). While some PGRPs possess catalytic activities, breaking the amide bond between MurNAc and L-alanine components of peptidoglycans, others are predicted to have receptor functions on the cell surface (Dziarski et al., 2003; Wang et al., 2003; Bastos et al., 2020). However, the specific functions of Acanthamoeba PGRPs remain to be elucidated.

## Sensing and signal transduction

**Figure 1:**
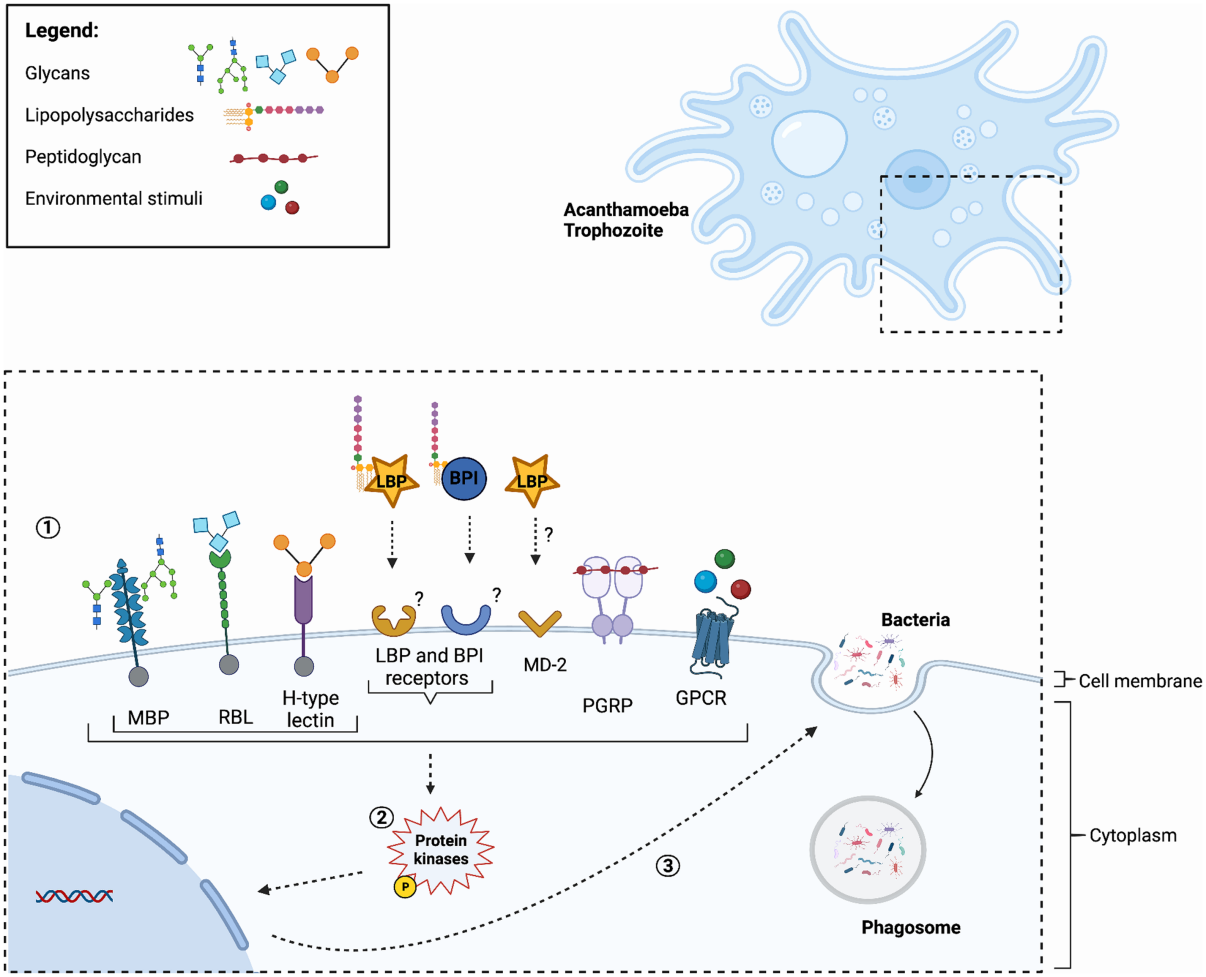
Hypothetical model of *Acanthamoeba* pathogen recognition receptors. (1) The process is initiated with the binding of Pattern Recognition Receptors (PRRs) to Microbe-Associated Molecular Patterns (MAMPs) or detection through *Acanthamoeba* cell surface receptors. Key components in this model include Mannose binding proteins (MBP), Rhamnose Binding Lectin (RBL) and H-type Lectins, which have a carbohydrate-recognition domain (CRD) and bind an array of glycans. Additionally, Lipopolysaccharide-Binding Proteins (LBPs), and Bactericidal/Permeability-Increasing Proteins (BPIs) are significant players. BPI and LBP often work together and are likely secreted. However, their respective receptors are yet to be identified. LBP may deliver bacterial components to the membrane bound myeloid differentiation factor 2 (MD-2) similar to that of the immune system of warm-blooded mammals. Peptidoglycan Recognition Proteins (PGRPs), likely target peptidoglycans found on Gram-positive bacteria. *Acanthamoeba* utilize various cell surface chemosensory receptors, particularly those of G-Protein-Coupled Receptors (GPCRs), to sense and respond to their environment. (2) These events trigger protein kinases phosphorylation, and subsequently. (3) This cascade of events leads to essential cellular processes, including phagocytosis. For a detailed depiction of the fate of bacteria following phagocytosis, readers are referred to a recent publication (Rayamajhee et al., 2022), where a comprehensive schematic is provided. Figure was created using Biorender (https://biorender.com/).

Recognition and interactions with prey requires sensing of signal cues. Acanthamoeba exhibit directed movement in response to an array of bacterial products (Schuster and Levandowsky, 1996). The displayed movement toward certain molecules hints at the presence of distinct receptors geared toward specific molecules. The *A. castellanii* Neff strain genome encodes multitude of receptors catering to extracellular cues, among these, the G-protein-coupled receptors (GPCRs), stand out as a prominent group (Clarke et al., 2013; Matthey-Doret et al., 2022). The genome sequence of *A castellanii* Neff strain also encodes multiple genes for phosphor-tyrosine signaling, mediated through a network of tyrosine kinases (PTKs), tyrosine phosphatases (PTPs), and proteins with Src homology 2 (SH2) domains (Clarke et al., 2013). Interestingly, the genome also encodes homologs of Dictyostelids atypical MAPK proteins ErkA and ErkB (Nichols et al., 2019). It is conceivable that a substantial subset of these receptors are dedicated to the vital task of sensing diverse food sources, including other microorganisms (Figure 1).

## H-type lectin domain proteins

The genome sequence of *A. castellanii* Neff strain revealed the presence of H-type lectin domain protein, a recently identified family of lectins which are known to specifically recognize glycosylated proteins containing GalNAc or galactose (Gal), these are associated with components of bacterial cell wall structures, such as LPS and peptidoglycans (Cummings et al., 2022). In other invertebrates, H-type lectins were speculated to be involved in antibacterial defense mechanism by aggregating bacteria (Mathieu et al., 2010). The family’s name originates from the quaternary structure of *Helix pomatia* agglutinin (HPA), which forms a hexamer (Sanchez et al., 2006). To get a better understanding of *A. castellanii* H-type lectin, we performed a multiple alignment of H-type lectin domains from, *A. castellanii* Neff strain (L8GSR8_ACACF), *H. pomatia* (Q2F1K8_HELPO) and *D. discoideum* [discoidins 1 (DIS1_DICDI) and II (DIS2_DICDII)] (Supplementary Figure S1). The amino acid residues crucial for ligand-binding, which were previously characterized in *H. pomatia* (Sanchez et al., 2006; Pietrzyk-Brzezinska and Bujacz, 2020), across all examined sequences were found to be conserved. This was also observed when predicted protein structures were aligned (Supplementary Figure S2). However, we note that *A. castellanii* H-type lectin domain shows some amino acid variation at these ligand-binding site, this divergence may potentially impact their binding specificities and/or functional characteristics. H-type lectins exhibit varying specificities for either αGalNAc or βGalNAc anomers (Sanchez et al., 2006), with exceptions to *D. discoideum* discoidins I and II which have the capacity to bind both anomers (Aragão et al., 2008). While it is suggestive that Acanthamoeba H-type lectin domain may also display a preference for GalNac anomers due to their similarities with Dictyosteleae, caution must be exercised in interpreting these findings and needs to be supported by experimental data.

## Role of flagellin in host interaction

Flagellin is a MAMP and serves as adhesins and plays a central role in the invasion of host cells (McSweegan and Walker, 1986; Nachamkin et al., 1993; Savelkoul et al., 1996; Feldman et al., 1998; Inglis et al., 2003). *O*-linked glycosylation of bacterial flagellin significantly affects flagellar assembly, motility, and host specificity. While motility was reported to be the driving force for interactions with Acanthamoeba, recent studies have revealed complexities. In the case of *C. jejuni*, flagellin glycosylation properties independent of motility was found to mediate interactions between the bacteria and Acanthamoeba (Nasher and Wren, 2023). This finding has added another layer of complexity, challenging traditional views on the role of motility in microbial interactions. It prompts questions about the specific molecular pathways and signaling mechanisms involved in the recognition and response to post translational modifications (PTM) of bacteria cell surface structures by Acanthamoeba. Additionally, it raises broader questions about the evolutionary significance of such adaptations and their implications for microbial ecology and host-microbe dynamics.

## Aggregation and phagocytosis

Some bacteria, such as *L. monocytogenes*, *Pseudomonas fluorescens*, and *C. jejuni* form aggregates on the surface on Acanthamoeba prior to phagocytosis (Preston and King, 1984; Axelsson-Olsson et al., 2005; Doyscher et al., 2013). For *L. moncytogenes*, this was proposed as a potential grazing strategy driven by Acanthamoeba locomotion to capture motile bacteria (de Schaetzen et al., 2022). This is consistent with the evolutionary adaptations that Acanthamoeba may have developed to flourish in their natural environment, potentially with the assistance of lectins. However, it is also worth considering that specific carbohydrate interactions driven equally by both organisms may contribute to bacterial aggregation, thereby fostering interactions between Acanthamoeba and microbial communities in their surroundings, and ultimately shaping microbial ecosystems. Indeed, this hypothesis gains further support from observed mutations at specific glycosylation sites on *C. jejuni* flagellin, which subsequently disrupts aggregation on the surface of Acanthamoeba (Nasher and Wren, 2023). On the other hand, it is worth noting that *C. jejuni* flagellin glycosylation is unique and therefore, this interaction may differ to other bacterial species.

## Concluding remarks

Bacterial encounters with Acanthamoeba have clearly shaped the evolution of both predator and prey, revealing why certain pathogens that mammals do not usually encounter (such as *Legionella*) are pre-armed in terms of survival in the hostile intracellular environments of warm-blooded animals (e.g., alveoli cells in the lung). This opportunistic nature of some pathogens makes sense when viewed from an eco-evo perspective (Pallen and Wren, 2007; Berlanga et al., 2021). Adapting to interact with a wide array of bacterial cell surface molecular structures, Acanthamoeba not only thrive but also play a pivotal role in shaping their intricate ecosystems. Despite their evolutionary divergence from warm-blooded host phagocytes, the conservation of innate immunity processes across distantly related organisms is evident. The interactions between Acanthamoeba and bacteria provide insights into microbial dynamics and immune responses.

## Limitations

Current research on Acanthamoeba-bacteria interactions, while insightful, has limitations. Most studies examine only a narrow range of bacterial species, which may not capture the full spectrum of interactions in natural environments. Additionally, findings from laboratory-cultured Acanthamoeba strains may not fully reflect the behavior of environmental isolates, potentially oversimplifying the complexity of these interactions. Furthermore, the molecular mechanisms behind Acanthamoeba recognition and response to various bacterial MAMPs remain only partially understood, necessitating further research.

## Future directions

Future research should address these limitations by expanding the range of bacterial species studied and incorporating environmental isolates to better replicate natural conditions. Investigating the complete diversity of bacterial interactions and their effects on bacterial transmission and pathogenicity could lead to new infection prevention and control strategies. Other amoeba such as the *D. discoideum* model can be used to understand these interactions in parallel. Key questions to understand the intricate mechanisms of immune recognition across the spectrum of life include:

- To what extent has the evolution of amoebae species been influenced by predator–prey relationships, and how does this impact their ecological niche and biodiversity?
- What molecular mechanisms underlie the specific recognition and response of Acanthamoeba to the diverse cell surface structures of bacteria, and how do these interactions shape microbial communities and ecological dynamics?
- How do the evolutionary adaptations of Acanthamoeba in recognizing and engulfing bacteria compare to immune responses in higher organisms, and what insights does this provide into the conservation of immune mechanisms across diverse life forms?

## Glossary

Acanthamoeba: Free-living amoebae belonging to the genus Acanthamoeba, which can act as predators of microorganisms, including bacteria
Capsular Polysaccharides (CPS): Polysaccharides that constitute the outermost layer of some bacteria, providing protection against immune responses
*Dictyostelid*: cellular slime molds are a group of social amoebae
Endosymbiosis: A symbiotic relationship in which one organism (the endosymbiont) lives within the cells or body of another organism
Flagellin: A protein component of bacterial flagella, recognized by host cells as a Microbe-Associated Molecular Pattern (MAMP)
Gram-Negative Bacteria: Bacteria with a cell wall structure that includes an outer membrane and a thinner layer of peptidoglycan
Gram-Positive Bacteria: Bacteria with a cell wall structure characterized by a thick layer of peptidoglycan
Lateral gene transfer (LGT): the process by which an organism incorporates genetic material from another organism without mating
Lipopolysaccharides (LPS): Complex molecules found in the outer membrane of Gram-negative bacteria, serving as MAMPs and endotoxins
Macrophages: Immune cells that engulf and digest pathogens
Mannose: A monosaccharide sugar of the aldohexose series of carbohydrates
Microbe-Associated Molecular Patterns (MAMPs): Conserved molecular structures on the surface of microorganisms recognized by host cells to initiate immune responses
*N*-linked glycosylation: The attachment of an oligosaccharide, a carbohydrate consisting of several sugar molecules, sometimes also referred to as glycan, to a nitrogen atom (the amide nitrogen of an asparagine (Asn) residue of a protein). *N*-linked glycosylation occurs widely in eukaryotes but rarely in bacteria
Neutrophils: White blood cells that are part of the immune system
*O*-linked glycosylation: is the attachment of a sugar molecule to the oxygen atom of serine (Ser) or threonine (Thr) residues in a protein. *O*-linked glycosylation occurs in both eukaryotes and prokaryotes
Pathogen recognition receptors (PRRs): are a class of germ line-encoded receptors that recognize microbe-associated molecular patterns
Phagocytes: Cells, such as macrophages, neutrophils, and amoebae, that are capable of engulfing and eliminating microorganisms
Post-Translational Modifications (PTMs): Chemical modifications made to proteins after they are synthesized, often affecting their function
Teichoic Acid (TA): A component of the cell walls of Gram-positive bacteria, including lipoteichoic acid (LTA)
