# Supplementary material for: Unravelling mechanisms of bacterial recognition by Acanthamoeba: insights into microbial ecology and immune responses
Source: Front Microbiol. 2024 Aug 23;15:1405133. doi: 10.3389/fmicb.2024.1405133 (PMC11377244; doi:10.3389/fmicb.2024.1405133)
Supplement: Supplementary file 1 [file Data_Sheet_1.PDF]

Supplementary File 1

Unravelling Acanthamoebae Mechanisms of Bacterial Recognition: Insights into Microbial Ecology and Immune Responses

Fauzy Nasher* and Brendan W Wren

Department of Infection Biology, London School of Hygiene and Tropical Medicine, London, United Kingdom

*Correspondence to:

Fauzy Nasher
E-mail: Fauzy.nasher1@lshtm.ac.uk

Sequence alignment of H-type lectin domains

```
L8GSR8_ACACF      -KGKGDRTFTTRVNFTSTGFATPPTVTLGLSAFDILDDS-DHRLALSLENVDKKGFTLNV
Q2F1K8_HELPO      PGRDNTRELAKNITFA-SPYCRPPVVLLSITQLDVEQSQ-NLRVIARLYSVSPTGFKASC
DIS1_DICDI        -TGSGKREVVVPVKFQ-FEFATLPKVALNFDQIDCTDATNQTRIGVQPRNITTKGFDCVF
DIS2_DICDI        -SGTGSRTIVRHVKFP-VEFLSVPIVSIGCKKVDAHTDNGQMRWEGKSENITTKGFDLTF
                      . * ..  :.*    :   * * :.   .*      : *      .:  .**

L8GSR8_ACACF      GTWLETKVWSLEVTWIAFDNS
Q2F1K8_HELPO      YTWHNTKVYSMSISWISIENY
DIS1_DICDI        YTWNENKVYSLRADYIATALE
DIS2_DICDI        ITWGNNAVYDLTFDYVAVEFN
                   ** :. *:.:   :::
```

Figure S1: Sequence alignment of H-type lectin domain from *Acanthamoebae castellanii* (L8GSR8_ACACF), *Helix pomatia* (Q2F1K8_HELPO) and *Dictyostelium discoideum* (dicsoidin I (DIS1_DICDI) and II (DIS2_DICDI)). Amino acids confirmed to be involved in ligand-binding [1] are highlighted; in red (fully conserved) and in green (conserved with slight variation). (*) indicates positions that are fully conserved; (:) indicated conserved between groups with similar properties; and (.) indicate conserved between groups of weakly similar properties.

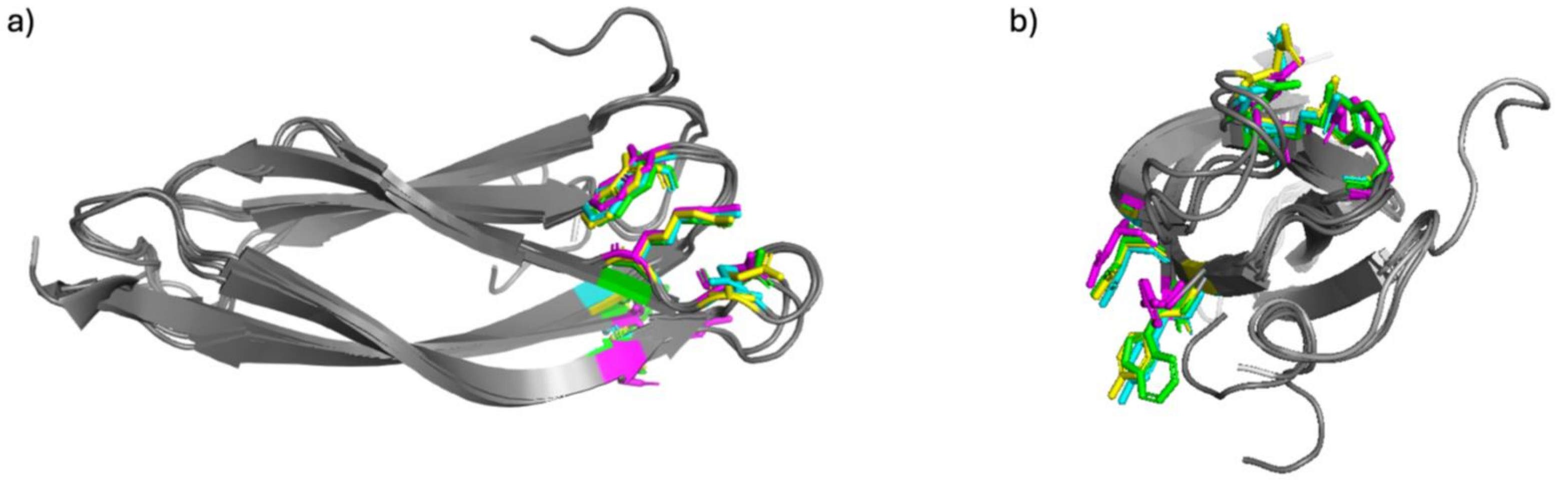

Figure S2: Protein Structure alignment of H-type lectin domain from *Acanthamoebae castellanii* (L8GSR8_ACACF), *Helix pomatia* (Q2F1K8_HELPO) and *Dictyostelium discoideum* (dicsoidin I (DIS1_DICDI) and II (DIS2_DICDI)). **a)** side view and **b)** top view. Predicted structures were acquired from Alpha fold [2] and aligned using PyMol (The PyMOL Molecular Graphics System, Version 3.0 Schrödinger, LLC.). Conserved amino acid are colored Green: L8GSR8_ACACF; Magenta: Q2F1K8_HELPO; Yellow: DIS1_DICDI and Cyan: DIS2_DICDI.

1. Pietrzyk-Brzezinska, A.J. and Bujacz, A. (2020) H-type lectins – Structural characteristics and their applications in diagnostics, analytics and drug delivery. *International Journal of Biological Macromolecules* 152, 735-747. https://doi.org/10.1016/j.ijbiomac.2020.02.320
2. Jumper, J. *et al.* (2021) Highly accurate protein structure prediction with AlphaFold. *Nature* 596, 583-589. 10.1038/s41586-021-03819-2
